# Molecular Characterization of Rifampicin-Resistant *Staphylococcus aureus* Isolates from Retail Foods in China

**DOI:** 10.3390/antibiotics10121487

**Published:** 2021-12-04

**Authors:** Jiahui Huang, Feng Zhang, Jumei Zhang, Jingsha Dai, Dongli Rong, Miao Zhao, Juan Wang, Yu Ding, Moutong Chen, Liang Xue, Qihui Gu, Shi Wu, Qingping Wu

**Affiliations:** 1 Guangdong Provincial Key Laboratory of Microbial Safety and Health, State Key Laboratory of Applied Microbiology Southern China, Key Laboratory of Agricultural Microbiomics and Precision Application, Ministry of Agriculture and Rural Affairs, Institute of Microbiology, Guangdong Academy of Sciences, Guangzhou 510070, China; jiahui0418@163.com (J.H.); zfloveissun@163.com (F.Z.); zhangjm926@126.com (J.Z.); daijs@gdim.cn (J.D.); rongdl69@126.com (D.R.); zm93032@163.com (M.Z.); wangjuan@scau.edu.cn (J.W.); dingyu@jnu.edu.cn (Y.D.); cmtoon@hotmail.com (M.C.); xueliang@gdim.cn (L.X.); guqh888@163.com (Q.G.); 2 School of Bioscience and Bioengineering, South China University of Technology, Guangzhou 510006, China

**Keywords:** *Staphylococcus aureus*, MRSA, rifampicin resistance, *rpoB* gene, MLST

## Abstract

This study investigated the molecular characteristics of rifampin-resistant (RIF-R) *Staphylococcus aureus* isolates recovered from 4300 retail food samples covering most provincial capitals in China, from 2011 to 2016. Of the 1463 *S. aureus* enrolled, 149 isolates (142 MSSA and 7 MRSA) were identified as rifampicin-resistant, including 20 high-level (MICs ≥ 8 μg/mL) and 129 low-level (MICs between 2 and 4 μg/mL) rifampicin-resistant strains. Most of the RIF-R *S. aureus* isolates were resistant to more than three antibiotics. The mutations in the rifampicin resistance-determining region of the *rpoB* gene were studied in all RIF-R strains. All of the strains presented the mutational change 481 His/Asn and five isolates presented an additional mutation, including 477 Asp/Tyr, 527 Ile/Met, and 466 Leu/Ser, respectively. Thirteen STs and twenty-one *spa* types were represented, in which five MRSA showed non-type SCC*mec* and the remaining MRSA belonged to SCC*mec* type IV—where, ST1-t127 was the predominant type from all of the isolates, while ST398-t034 was the predominant type for the MRSA isolates. In this study, we found that the food-related RIF-R *S. aureus* may have a unique genetic background selection. However, the scenario regarding the presence of RIF-R *S. aureus,* especially MRSA, in retail food in China is not favorable and warrants public attention.

## 1. Introduction

*Staphylococcus aureus* is a versatile pathogens capable of causing nosocomial and community-acquired infections [1]. It is one of the most important foodborne microorganisms, for which its pathogenicity affects human’s health through staphylococcal enterotoxins (SEs). The first food poisoning event caused by *staphylococci* occurred in 1884 [2]. Recently, it has been found in food producing chains, for example the equipment used for the production of food and in contaminated ingredients and so on [3,4]. Between 2000 and 2015, *S. aureus* was one of the most frequently reported pathogens in ready-to-eat (RTE) food (such as cheeses, cured meats, sausages, smoked fishes, salads, and so on) in Africa and Europe [5,6,7]. In China, the prevalence of *S. aureus*-contaminated retail food increased over time [8].

Rifampicin (RIF) is the most used broad-spectrum antibiotic against bacterial pathogens. It has a potent bactericidal activity, good tissue penetration and modest activity against non-growing cells [9]. RIF is currently indicated in combination therapy for implant-associated infections, serious *S. aureus* infections, and has been shown to broaden the coverage of vancomycin-intermediate *S. aureus* (VISA) when used in combination with linezolid or daptomycin [10]. However, the long-term use of RIF, as well as, its use as a monotherapy, has been shown to be associated with the development of RIF-resistant (RIF-R) *S. aureus*. Nowadays, many researchers have suggested that the major reason for RIF resistance is the mutations, mainly single nucleotide polymorphisms (SNPs), in the *rpoB* gene shown in the *Staphylococcus* species [11,12,13]. One of the first report suggested that *rpoB* gene mutations involving eight conserved amino acids were identified in RIF-R *Mycobacterium tuberculosis*. All mutations were clustered within a region of 23 amino acids [14]. This conclusion was further verified in RIF-resistance *Staphylococci* [11,15]. The β-subunit of the bacterial RNA polymerase, encoded by the *rpoB* gene, plays an important role in RIF inhibition [11]. In *S. aureus*, a number of studies have revealed a worrying link between certain *rpoB* mutations and decreased susceptibility not only to RIF, but also to other last-line anti-MRSA antibiotics such as vancomycin, daptomycin, β-lactams, and imipenem [16,17,18,19,20].

In our previous study, we collected 4300 retail food samples from supermarkets, fairs, and farmers’ markets. We covered most of the provincial capitals of China from July 2011 to June 2016 and collected 1463 *S. aureus* isolates from 1063 positive samples from all of the sampling sites [21,22]. Furthermore, most of *S. aureus* isolates from retail food showed severe antibiotic resistance [22,23,24,25]. However, there were a limited number of reported studies on RIF-R *S. aureus* isolated from retail food. In order to better understand the epidemic background and the molecular characters in RIF-R isolates for *S. aureus* in food, this study re-identified the RIF-R *S. aureus* in food-related isolates and further studied RIF-R *S. aureus* in terms of the following: (1) the mutations in the *rpoB* gene that contributed to RIF resistance, and (2) the antimicrobial resistance profiles and their genotypic types (MLST and *spa* typing).

## 2. Results

### 2.1. Distribution of Rifampicin-Resistant S. aureus from Retail Foods in China

In total, 149 RIF-R *S. aureus* isolates, including 7 MRSA strains, were re-identified by the disk diffusion method and were used for the present study. The RIF-R *S. aureus* isolates represented 10.2% of all *S. aureus* isolates. The origin of the strains was mainly from raw meat, aquatic products, quick-frozen products, and RTE food, including 35 (8.4%) of the 419 isolates from raw meat, 45 (9.5%) of the 473 isolates from aquatic products, 43 (12.0%) of the 358 isolates from quick-frozen products, 22 (16.8%) of the 131 isolates from ready-to-eat food, 1 (2.4%) of the 41 isolates from edible mushrooms, and 3 (25.0%) of the 12 isolates from pasteurized milk, whereas vegetables were free of RIF-R *S. aureus* isolates (Table 1). The RIF-R MRSA isolates were distributed in raw meat (n = 4), aquatic products (n = 1), quick-frozen products (n = 1), and RTE food (n = 1). The prevalence of RIF-R MSSA in foods was significantly higher than RIF-R MRSA (*p* < 0.001, χ^2^ test).

### 2.2. Antibiotic Resistance of Rifampicin-Resistant S. aureus Isolates

The antibiotic susceptibility results of 149 RIF-R *S. aureus* isolates are shown in Table 2. In this study, most of RIF-R *S. aureus* isolates were resistant to more than three antibiotics, whereas only 9.4% of isolates were resistant to 0–3 antibiotics. For multidrug resistance isolates, 77.9% of isolates were resistant to 4–10 antibiotics, 10.7% of isolates were resistant to 11–15 antibiotics, and 2.0% of isolates were resistant to 16–24 antibiotics. The isolates were susceptible to linezolid and teicoplanin, and the frequencies of resistance to individual agents were 94.6% for ampicillin and 92.6% for penicillin G, followed by 75.8% for kanamycin, 53.7% for erythromycin, 49% for tetracycline, 30.2% for clindamycin, 28.9% for gentamycin, 26.8% for telithromycin, 26.2% for norfloxacin, 24.8% for fusidic acid, 18.1% for ciprofloxacin, 16.1% for streptomycin, 10.7% for chloramphenicol, 6.7% for amikacin, 6.7% for trimethoprim/sulphamethoxazole 1:19, 5.4% for ceftazidime, 4.7% for cefoxitin, 4.0% for cefepime, 3.4% for quinupristin/dalfopristin, and 2.0% for nitrofurantoin. Only seven RIF-R *S. aureus* isolates belonged to MRSA, whereas most of RIF-R *S. aureus* isolates were MSSA (Table 1). Interestingly, the RIF-R MRSA isolates showed multi-drug resistance to more than 10 antibiotics in 71.4% of cases (five out of seven isolates). Rates of resistance to AMC, FEP, CAZ, GEN, CHL, CLI, TEL, SXT, and NIT, and intermediate resistance to RIF were all significantly higher in the MRSA group than in the MSSA group (*p* < 0.01, χ^2^ test) (Table 2). In total, the MDR rate for MRSA isolates was significantly higher than that for MSSA (*p* < 0.01, χ^2^ test).

### 2.3. Rifampicin Resistance Levels and Associated rpoB Mutations

Among the 149 RIF-R *S. aureus* isolates, 129 isolates showed a low level of RIF resistance (MICs between 2 and 4 μg/mL) and 20 isolates showed a high level of RIF resistance (MICs ≥ 8 μg/mL). In addition, three out of seven MRSA showed a high level of RIF resistance (MIC > 16 μg/mL). The mutations in the RIF resistance-determining region of the *rpoB* gene were studied in all 149 RIF-R strains. The results are shown in Table 3. All 149 strains presented the mutational change 481 His/Asn, determined by a mutation in cluster I of the *rpoB* gene, conferring a low-level of RIF resistance. Three additional mutations were found in five isolates, including 477 Asp/Tyr (n = 3), 527 Ile/Met (n = 1), and 466 Leu/Ser (n = 1). Of these, two isolates with MIC ≥ 16 μg/mL had an additional amino acid substitution: 466 Leu/Ser (n = 1) or 527 Ile/Leu (n = 1), associated with a high level of RIF resistance.

### 2.4. Distribution of Molecular Types

MLST and *spa* typing were carried out in all 149 RIF-R *S. aureus* strains. The results are shown in Table 4. Among these isolates, a total of 13 STs and 21 *spa* types were represented (except sta43-0 and sta43-2 were shown to be on-type). The predominant MLST types were ST1 (97/149, 65.1%), followed by ST72 (15/149, 10.1%), ST5 (11/149, 7.4%), ST398 (10/149, 6.7%), ST12 (5/149, 3.4%), and ST45 (4/149, 2.7%), and the seven singletons included ST25, ST59, ST2592, ST4449, ST4470, ST4472, and ST9. The most commonly observed *spa* type was t127 (90/149, 60.4%), followed by t3092 (15/149, 10.1%), t002 (9/149, 6.0%), t034 (9/149, 6.0%), t213 (4/149, 2.7%), t116 (4/149, 2.7%), t177 (2/149, 1.3%), and t5500 (2/149, 1.3%), and the 12 singletons included t1381, t17635, t1908, t2207, t2459, t2720, t559, t9472, t17632, t17887, t163, and t899. There were 27 different ST-*spa* types in this research combining the STs and *spa* types, of which ST398-t034 was the predominant type (3/7, 42.9%) of MRSA isolate, whereas ST1-t127 was the predominant type (96/142, 67.6%) of MSSA isolate in RIF-R *S. aureus* (Table 4). Only ST1 MRSA (n = 1) and ST45 MRSA (n = 1) harbored SCC*mec* type IV.

## 3. Discussion

In recent years, the frequency of the RIF-R *S. aureus* isolated from clinics in China has increased rapidly, showing that the percentage of RIF-R MRSA isolates was only 15.5% in 2004, but increased rapidly to 50.2% in 2008 [26,27]. However, studies on RIF-R *S. aureus* isolated from retail food have rarely been reported in China. In this study, a total of 149 food-related *S. aureus* isolates (10.2%) were identified as RIF-R isolates, which covered all types of animal-derived food, including raw meat, aquatic products, quick-frozen products, and RTE food. Recently, Şanlıbaba P. (2022) collected 96 *S. aureus* strains from retail raw beef, sheep and lamb meat in Turkey, and 8.33% of strains were found to be resistant to RIF [28]. In Portugal, only one RIF-R *S. aureus* was detected in various foods [29]. In north-western Greece, only 1.6% RIF-R *S. aureus* was found in dairy products [30]. Compared with these studies, the prevalence rate of RIF-R *S. aureus* isolates in food the food we examined in China was not low, which should be brought to public attention.

RIF acts by interacting specifically with the β-subunit of the bacterial RNA polymerase encoded by the *rpoB* gene [31,32]. Many previous studies have reported that the mutations of *rpoB* were a response to RIF resistance in bacteria such as *Escherichia coli* [33], *Mycobacterium leprae* [34], *Streptococcus pneumoniae* [35], and *Neisseria meningitides* [36]. In this study, we also amplified and sequenced the portions of *rpoB* of RIF-R *S. aureus* isolates in food. It was found that all strains presented the mutational change 481 His/Asn. In China, Zhou et al. (2012) collected 88 RIF-R MRSA isolates from clinical specimens in the Microbiology Department of Anhui Provincial Hospital in China, and found that the mutation at 481 His/Asn was the most common in 95.5% of RIF-R isolates [37]. Similarly, this is consistent with Liang’s research, showing 481 His/Asn mutations of *rpoB* in all RIF-R or intermediate-resistant isolates from women and children in Guangzhou, China [38]. Interestingly, as described in previous literature, the position of 481 RpoB presents a hot-spot for amino acid residue replacement and has recently been found to be the strongest genetic marker of increased vancomycin resistance [26,39,40]. Therefore, the scenario regarding the presence of RIF-R *S. aureus,* especially MRSA in retail food in China, should warrant public attention. Moreover, as previously described, a high-level resistance of RIF may also be attributed to additional mutations within *rpoB* [41]. Isolates containing multiple mutations also showed high-level RIF resistance [42]. According to Zhou et al. (2012), it was found that 87.5% RIF-R *S. aureus* had an additional mutation 466 Leu/Ser and the RIF resistance level was higher [37]. In our study, fewer strains had additional mutations, such as 527 Ile/Met and 466 Leu/Ser mutations, which was also associated with a higher-level of resistance of RIF. Although the rate of multiple mutations was not high, it should be paid attention.

In this study, it was found that most RIF-R *S. aureus* isolated from retail food belonged to ST1-t127. Meanwhile, ST398-t034 was the predominant type of MRSA isolate. This is different from the predominant types of food-related MRSA, which, when isolated simultaneously with RIF-R strains, showed that the predominant type was ST59-t437 [22]. According to previous studies, the predominant types of clinical RIF-R strains were ST239 or ST45 [37,38,43,44]. In 2018, Liang et al. collected 131 *S. aureus* isolated from patients in four centers for women and children in Guangzhou, and found that CC59 (ST59-IV, 48.8%; ST338-III, 35.7%) was a major clone (84.4%) among the MRSA isolates, but 93.8% of the RIF-R or intermediate resistant isolates belonged to ST45 [38]. Thus, the RIF-R *S. aureus* in the food may have a unique genetic background selection. However, the reasons for this specific selection need to be further explored.

As we know, RIF is an important antibiotic used in combination therapy for the treatment of deep-seated staphylococcal infections [45]. This review also provided evidence that RIF combined with trimethoprim/sulfamethoxazole and minocycline is associated with a high rate of isolation of *S. aureus* resistance, whereas RIF combined with other agents is associated with lower rates of resistance [46]. Furthermore, a combination therapy, with an antibiotic such as vancomycin, is often required in order to reach deep-seated infections effectively [47]. Thus, resistance to RIF can make the treatment of *S. aureus* infection, especially MRSA infection, more difficult. Except for RIF, antibiotics such as ampicillin, penicillin, kanamycin, erythromycin, and tetracycline, are common drugs used for *S. aureus* infection [48]. However, the resistance rates of these antibiotics for RIF-R *S. aureus* exceeded 50% in this study. Moreover, most of the RIF-R *S. aureus* isolates were resistant to more than three antibiotics. Of which, 19 RIF-R *S. aureus* isolates were resistant to more than 10 antibiotics, namely 5 MRSA and 14 MSSA. Interestingly, ST398 MRSA and MSSA (36.8%, 7/19) were the predominant types of multi-drug resistant isolates in RIF-R *S. aureus*. As we know, MRSA CC398 is a frequent colonizer of humans with occupational exposure to livestock [49]. Recently, infections caused by MSSA ST398 have been described in humans [50,51,52] and it has been shown to cause infections more frequently than MRSA ST398 [21]. In 2011, Argudín et al. studied the resistance determinants of German *S. aureus* ST398 isolates from non-human sources, and found that none of the isolates were resistant to RIF [53]. Thus, further investigation is required to elucidate the emergence of multidrug-resistant ST398 in RIF-R *S. aureus*.

## 4. Materials and Methods

### 4.1. Bacterial Strains

A total of 1463 *S. aureus* were isolated from 4300 retail food products between July 2011 and June 2016. The samples were collected from supermarkets, fairs, and farmers’ markets, which covered most of the provincial capitals of China in 39 cities (Figure S1). One hundred samples were collected from each city, except for Guangzhou, where 500 samples were collected. These strains were isolated from seven types of food products, including 419 isolates from meat and meat products (bacon/sausage, poultry, pork, mutton, and beef), 473 isolates from aquatic products (freshwater fish, shrimp, and seafood), 358 isolates from quick-frozen products (frozen dumplings/steamed stuffed buns and frozen meat), 131 isolates from ready-to-eat (RTE) food (cold vegetable/noodle dishes in sauce, fried rice/sushi, roast meat, sausage, and ham), 41 isolates from edible mushrooms, 29 isolates from vegetables, and 12 isolates from pasteurized milk. These isolates were obtained according to the GB 4789.10-2010 food microbiological examination of *S. aureus* (National Food Safety Standards of China) and the most probable number (MPN) method [54]. The *S. aureus* isolates were streaked onto chromogenic *S. aureus* agar plates (Huankai) and were re-identified by Gram’s staining, microscopic examination, coagulase testing, and catalase testing. MRSA was initially screened by the cefoxitin disk diffusion method, and then confirmed by polymerase chain reaction (PCR) detecting *mecA*.

### 4.2. Antimicrobial Susceptibility Testing

All isolates were tested for antimicrobial susceptibility by the disk diffusion method using Mueller−Hinton agar and commercially available discs (Oxoid, Basingstoke, UK). The antimicrobial agents used were amoxycillin/clavulanic acid (30 µg), ampicillin (10 µg), cefepime (10 µg), cefoxitin (30 µg), penicillin G (10 U), ceftazidime (30 µg), amikacin (30 µg), gentamicin (10 µg), kanamycin (30 µg), streptomycin (25 µg), chloramphenicol (30 µg), clindamycin (2 µg), erythromycin (15 µg), telithromycin (15 µg), ciprofloxacin (5 µg), norfloxacin (10 µg), tetracycline (30 µg), linezolid (30 µg), trimethoprim/sulphamethoxazole 1:19 (25 µg), rifampicin (5 µg), quinupristin/dalfopristin (15 µg), teicoplanin(30 µg), nitrofurantoin (300 µg), and fusidic acid (10 µg). *Staphylococcus aureus* ATCC25923 and *Escherichia coli* ATCC25922 were used as the quality control organisms [55]. The MICs of rifampicin for all *S. aureus* isolates were further determined using the agar dilution method. The results of the antimicrobial susceptibilities of the analyzed strains were scored according to the guidelines of the CLSI [55].

### 4.3. Detection of Rifampicin Resistance-Associated Mutations

All of the isolates were incubated at 37 °C overnight on BHI (brain heart infusion broth). Genomic DNA was extracted using a Genomic DNA Extraction Kit (Magen Biotech, Guangzhou, China) according to the manufacturer’s instructions. The concentration of the genomic DNA was determined at 260 nm using a NanoDrop ND-1000 UV−VIS spectrophotometer (Thermo Fisher Scientific, Waltham, MA, USA). An internal sequence of gene *rpoB* of 432 bp (nucleotides 1216 to 1648) was amplified by PCR. This region included the RIF resistance determining cluster I (nucleotides 1384–1464, amino acid number 462–488) and cluster II (nucleotides 1543–1590, amino acid number 515–530). The amplification was carried out in 149 RIF-R strains. Amplification was carried out as previously described [56]. The PCR products were purified and analyzed by DNA sequencing. The nucleotide sequences obtained were compared to the *rpoB* wild type sequence from *S. aureus* subsp. aureus (GenBank accession number: X64172) using the clustalw software (http://www.ebi.ac.uk/tools/clustalw/index.html, accessed 5 June 2019).

### 4.4. Molecular Typing

#### 4.4.1. *spa*-Typing

Sequence typing of the *S. aureus* protein A (*spa*) repeat region was amplified according to a published protocol [57]. All isolates were analyzed using the primers spa-1113f (5′-TAAAGACGATCCTTCGGTGAGC-3′) and spa-1514r (5′-CAGCAGTAGTGCCGTTTGCTT-3′). The PCR amplification conditions were as follows: an initial cycle of 80 °C for 5 min; 35 cycles of 94 °C for 45 s, 60 °C for 45 s, 72 °C for 2 min, and a final extension at 72 °C for 10 min. The *spa* types were randomly assigned using the Spa Server website (http://spaserver2.ridom.de, accessed 10 July 2019).

#### 4.4.2. Multilocus Sequencing Typing

The MLST scheme used to characterize *S. aureus* isolates is based on the sequence analysis of the following seven housekeeping genes: *arcC* (Carbamate kinase), *aroE* (Shikimate dehydrogenase), *glpF* (Glycerol kinase), *gmk* (Guanylate kinase), *pta* (Phosphate acetyltransferase), *tpi* (Triosephosphate isomerase), and *yqil* (Acetyle coenzyme A acetyltransferase) [58]. The PCR amplification conditions were as follows: an initial cycle of 94 °C for 5 min, 35 cycles of 94 °C for 30 s, 55 °C for 30 s, 72 °C for 2 min, and a final extension at 72 °C for 10 min. The DNA fragments were purified using a PCR purification kit (Qiagen, Hilden, Germany) and were sequenced in each direction with Big Dye fluorescent terminators on an ABI 3730XL sequencer (Applied BioSystems, Foster, USA). For each MLST locus, an allele number was given to each distinct sequence variant, and a distinct sequence type (ST) number was attributed to each distinct combination of alleles at the seven genes. Sequence types (STs) were determined by using the *Staphylococcus aureus* MLST database (https://pubmlst.org/saureus/, accessed 20 July 2019). Sequence Type Analysis and Recombinational Tests software (S.T.A.R.T. ver.2; http://pubmlst.org/software/analysis/start2, accessed 25 July 2019) was used to analyze the data of MLST.

#### 4.4.3. SCC*mec* Typing

The SCC*mec* typing method was performed on the isolates by multiplex PCR, as previously described [59]. The PCR amplification conditions had an initial denaturation step at 94 °C for 5 min, followed by 10 cycles of 94 °C for 45 s, 65 °C for 45 s, and 72 °C for 1.5 min and another 25 cycles of 94 °C for 45 s, 55 °C for 45 s, and 72 °C for 1.5 min, ending with a final extension step at 72 °C for 10 min and followed by a hold at 4 °C. All PCR assay runs incorporated a reagent control (without template DNA). The PCR amplicons were visualized using a UV light box after electrophoresis on a 2% agarose gel containing 0.5 g/mL ethidium bromide.

### 4.5. Statistical Analysis

Statistical analyses were performed using SPSS 17.0 Data Editor (SPSS, Inc., Chicago, IL, USA). Categorical variables were described using frequencies and their proportion, and were compared using the Chi-square (χ^2^) test. *p* < 0.05 was considered to indicate a statistically significant difference.

## 5. Conclusions

In summary, we investigated food-related RIF-R *S. aureus* and determined its genetic background in China in this study. RIF-R *S. aureus* isolates were observed in most animal-derived types of food samples and rare in other types of food. Most of the RIF-R *S. aureus* isolates showed multiple resistance and the reason for RIF resistance in *S. aureus* is closely associated with the mutations that occur in the *rpoB* gene. This study indicated that the RIF-R *S. aureus* in food may have a unique genetic background selection. However, monitoring of the emergence and possible dissemination of the MRSA ST398 strains among food-related infections for RIF treatment is warranted.

## Figures and Tables

**Table 1:** Distributions of RIF-R *S. aureus* from retail foods in China.

Types of Product	No. of *S. aureus*	No. (%) of RIF-R *S. aureus* Isolates	No. (%) of RIF-R MRSA	No. (%) of RIF-R MSSA	*p*-Value ^a^
Raw meat	419	35 (8.4)	4 (1.0)	31 (7.4)	<0.01
Aquatic products	473	45 (9.5)	1 (0.2)	44 (9.3)	<0.01
Quick-frozen meat	358	43 (12.0)	1 (0.3)	42 (11.7)	<0.01
Ready-to-eat food	131	22 (16.8)	1 (0.8)	21 (16.0)	<0.01
Edible mushrooms	41	1 (2.4)	0 (0.0)	1 (2.4)	<0.01
Vegetables	29	0 (0.0)	0 (0.0)	0 (0.0)	NA ^b^
Pasteurized milk	12	3 (25.0)	0 (0.0)	3 (25.0)	<0.01
Total	1463	149 (10.2)	7 (0.5)	142 (9.7)	<0.01

^a^ RIF-R MRSA vs. MSSA by the Chi-squared test (two-sided); ^b^ NA—not applicable.

**Table 2:** Results of antimicrobial susceptibility tests of 149 RIF-R *S. aureus* isolates obtained from retail food in China.

Antibiotics ^a^	RIF-R *S. aureus* (n = 149)		RIF-R MRSA (n = 7)		RIF-R MSSA (n = 142)		*p*-Value ^b^
	NO. of R (%)	NO. of I (%)	NO. of R (%)	NO. of I (%)	NO. of R (%)	NO. of I (%)	
AMC	10 (6.7)	-	6 (85.71)	-	4 (2.82)	-	<0.01
AMP	141 (94.6)	-	7 (100)	-	134 (94.37)	-	1.000
FEP	6 (4.0)	2 (1.3)	5 (71.43)	2 (28.57)	1 (0.7)	0 (0.0)	<0.01
FOX	7 (4.7)	-	7 (100)	-	0 (0.0)	-	NA
PEN	138 (92.6)	-	7 (100)	-	131 (92.25)	-	0.980
CAZ	8 (5.4)	23 (15.4)	7 (100)	0 (0.0)	1 (0.7)	23 (16.20)	<0.01
AMK	10 (6.7)	51 (34.2)	1 (14.29)	3 (42.86)	9 (6.34)	48 (33.80)	0.877
GEN	43 (28.9)	0 (0.0)	5 (71.43)	0 (0.0)	38 (26.76)	0 (0.0)	<0.01
KAN	113 (75.8)	11 (7.4)	5 (71.43)	1 (14.29)	108 (76.06)	10 (7.04)	1.000
STR	24 (16.1)	103 (69.1)	4 (57.14)	2 (28.57)	20 (14.08)	101 (71.13)	0.398
CHL	16 (10.7)	27 (18.1)	4 (57.14)	0 (0.0)	12 (8.45)	27 (19.01)	<0.01
CLI	45 (30.2)	16 (10.7)	6 (85.71)	0 (0.0)	39 (27.46)	16 (11.27)	<0.01
ERY	80 (53.7)	16 (10.7)	7 (100)	0 (0.0)	73 (51.41)	16 (11.27)	0.069
TEL	40 (26.8)	27 (18.1)	6 (85.71)	1 (14.29)	34 (23.94)	26 (18.31)	<0.01
CIP	27 (18.1)	31 (20.8)	4 (57.14)	1 (14.29)	23 (16.20)	30 (21.13)	0.034
NOR	39 (26.2)	11 (7.4)	4 (57.14)	0 (0.0)	35 (24.65)	11 (7.75)	0.190
TET	73 (49.0)	5 (3.4)	5 (71.43)	0 (0.0)	68 (47.89)	5 (3.52)	0.461
LZD	0 (0.0)	-	0 (0.0)	-	0 (0.0)	-	NA
RIF	122 (81.9)	27 (18.1)	4 (57.14)	3 (42.86)	118 (83.10)	24 (16.90)	0.216 ^c^
SXT	10 (6.7)	2 (1.3)	4 (57.14)	0	6 (4.23)	2 (1.41)	<0.01
QD	5 (3.4)	11 (7.4)	0 (0.0)	1 (14.29)	5 (3.52)	10 (7.04)	NA
TEC	0 (0.0)	42 (28.2)	0 (0.0)	2(28.57)	0 (0.0)	40 (28.17)	NA
NIT	3 (2.0)	18 (12.1)	2 (28.57)	0 (0.0)	1 (0.7)	18 (12.68)	<0.01
FD	37 (24.8)	-	2 (28.57)	0 (0.0)	35 (24.65)	0 (0.0)	1.000
0–3 Antimicrobial	14 (9.4)		0(0.0)		14 (9.9)		-
4–10 Antimicrobial	116 (77.9)		2 (28.6)		114 (80.2)		-
11–15 Antimicrobial	16 (10.7)		2 (28.6)		14 (9.9)		-
16–24 Antimicrobial	3 (2.0)		3 (42.8)		0 (0.0)		-

R: Resistant; I: Intermediate; NA: not applicable. ^a^ AMC: Amoxicillin/clavulanic acid; AMP: Ampicillin; FEP: Cefepime; FOX: Cefoxitin; PEN: Penicillin G; CAZ: Ceftazidime; AMK: Amikacin; GEN: Gentamicin; KAN: Kanamycin; STR: Streptomycin; CHL: Chloramphenicol; CLI: Clindamycin; ERY: Erythromycin; TEL: Telithromycin; CIP: Ciprofloxacin; NOR: Norfloxacin; TET: Tetracycline; LZD: Linezolid; RIF: Rifampicin; SXT: Trimethoprim/sulphamethoxazole 1:19; QD: Quinupristin/dalfopristin; TEC: Teicoplanin; NIT: Nitrofurantoin; FD: Fusidic acid; ^b^ Antibiotic resistance of MRSA vs. MSSA using the Chi-squared test (two-sided). ^c^ Rifampicin-intermediate of MRSA vs. MSSA using the Chi-squared test (two-sided).

**Table 3:** The characteristics of the RIF-R *S. aureus* isolates studied.

Nucleotide Mutation	Amino Acid Substitution	Mutation Types	RIF MIC (μg/mL)	Number of Isolates
CAT → AAT	His481/Asn	cluster I	2	77
GCA → ACA, CAT → AAT	Asp473/Tyr, His481/Asn	cluster I	2	1
CAT → AAT	His481/Asn	cluster I	4	51
GCA → ACA, CAT → AAT	Asp473/Tyr, His481/Asn	cluster I	4	2
CAT → AAT	His481/Asn	cluster I	8	10
CAT → AAT	His481/Asn	cluster I	16	3
CAT → AAT	His481/Asn	cluster I	>16	5
CAT → AAT, TAA → GAA	His481/Asn, Ile527/Met	cluster I, cluster II	>128	1
CAT → TAT, CAT → AAT	Leu466/Ser, His481/Asn	cluster I	>128	1

**Table 4:** Molecular features of RIF-R *S. aureus* isolates.

STs (No.)	*spa* Types (No.)	Nucleotide Mutation	Amino Acid Substitution	Mutation Types
ST1 (97)	t127 (82)	CAT → AAT	His481/Asn	cluster I
	t1381 (1)	CAT → AAT	His481/Asn	cluster I
	t17635 (1)	CAT → AAT	His481/Asn	cluster I
	t177 (2)	CAT → AAT	His481/Asn	cluster I
	t1908 (1)	CAT → AAT	His481/Asn	cluster I
	t2207 (1)	CAT → AAT	His481/Asn	cluster I
	t2459 (1)	CAT → AAT	His481/Asn	cluster I
	t2720 (1)	CAT → AAT	His481/Asn	cluster I
	t5500 (2)	CAT → AAT	His481/Asn	cluster I
	t559 (1)	CAT → AAT	His481/Asn	cluster I
	t127 (1)	CAT → TAT, CAT → AAT	Leu466/Ser, His481/Asn	cluster I
	t127 (3)	GCA → ACA, CAT → AAT	Asp473/Tyr, His481/Asn	cluster I
ST72 (15)	t3092 (15)	CAT → AAT	His481/Asn	cluster I
ST5 (11)	t002 (9)	CAT → AAT	His481/Asn	cluster I
	non type (2)	CAT → AAT	His481/Asn	cluster I
ST398 (10)	t034 (9)	CAT → AAT	His481/Asn	cluster I
	t9472 (1)	CAT → AAT	His481/Asn	cluster I
ST12 (5)	t213 (4)	CAT → AAT	His481/Asn	cluster I
	t17632 (1)	CAT → AAT	His481/Asn	cluster I
ST45 (4)	t116 (4)	CAT → AAT	His481/Asn	cluster I
ST25 (1)	t17887 (1)	CAT → AAT	His481/Asn	cluster I
ST59 (1)	t163 (1)	CAT → AAT	His481/Asn	cluster I
ST2592 (1)	t127 (1)	CAT → AAT	His481/Asn	cluster I
ST4449 (1)	t127 (1)	CAT → AAT	His481/Asn	cluster I
ST4470 (1)	t127 (1)	CAT → AAT	His481/Asn	cluster I
ST4472 (1)	t127 (1)	CAT → AAT	His481/Asn	cluster I
ST9 (1)	t899 (1)	CAT → AAT, TAA → GAA	His481/Asn, Ile527/Met	cluster I, cluster II

## Supplementary Materials

The following are available online at https://www.mdpi.com/article/10.3390/antibiotics10121487/s1, Figure S1: The locations of the sampling sites for this study in China.
