# Supplementary figures and images for: Molecular Characterization of Rifampicin-Resistant Staphylococcus aureus Isolates from Retail Foods in China
Source: Antibiotics (Basel). 2021 Dec 4;10(12):1487. doi: 10.3390/antibiotics10121487 (PMC8698944; doi:10.3390/antibiotics10121487)

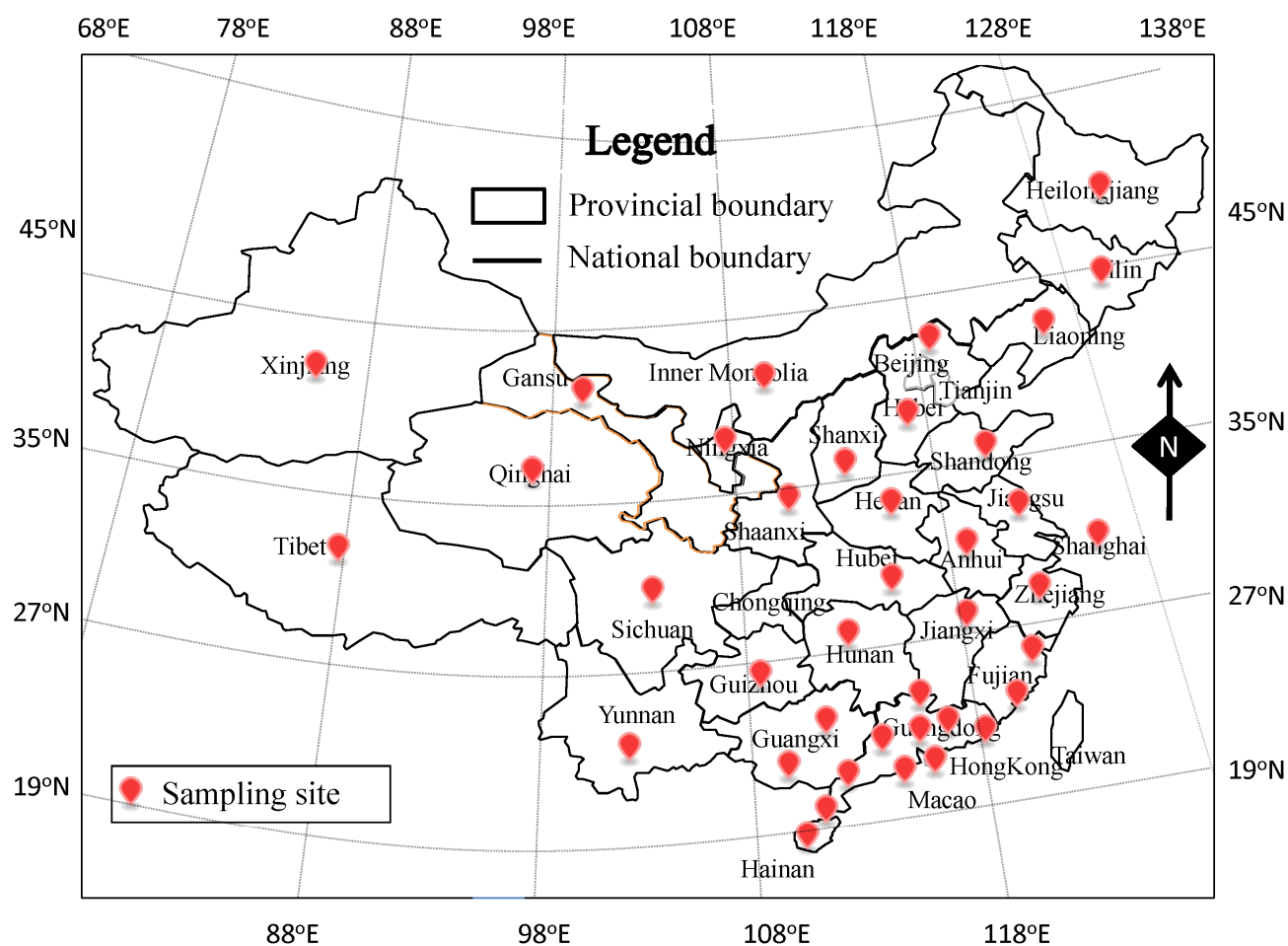

**Figure S1.** The locations of the sampling sites for this study in China.

Supplement: Supplementary file 1 [file antibiotics-10-01487-s001.zip › antibiotics-1451076-supplementary.pdf]
